# Digoxin Induces Human Astrocyte Reaction In Vitro

**DOI:** 10.1007/s12035-022-03057-1

**Published:** 2022-10-12

**Authors:** David Pamies, Tatjana Vujić, Domitille Schvartz, Julien Boccard, Cendrine Repond, Carolina Nunes, Serge Rudaz, Jean-Charles Sanchez, Víctor González-Ruiz, Marie-Gabrielle Zurich

**Affiliations:** 1 Department of Biological Sciences, University of Lausanne, Lausanne, Switzerland; 2 Swiss Centre for Applied Human Toxicology (SCAHT), Basel, Switzerland; 3 Translational Biomarker Group, Department of Medicine, University of Geneva, Geneva, Switzerland; 4 School of Pharmaceutical Sciences and Institute of Pharmaceutical Sciences of Western Switzerland, University of Geneva, Geneva, Switzerland

**Keywords:** Neurotoxicity, Astrocytes, Astrogliosis, Glycolysis, Energy metabolism, Neuroinflammation

## Abstract

Astrocyte reaction is a complex cellular process involving astrocytes in response to various types of CNS injury and a marker of neurotoxicity. It has been abundantly studied in rodents but relatively poorly in human cells due to limited access to the brain. Astrocytes play important roles in cerebral energy metabolism and are also key players in neuroinflammation. Astroglial metabolic and inflammatory changes have been reported with age, leading to the hypothesis that mitochondrial metabolism and inflammatory responses are interconnected. However, the relationship between energy metabolism and astrocyte reactivity in the context of neurotoxicity is not known. We hypothesized that changes in energy metabolism of astrocytes will be coupled to their activation by xenobiotics. Astrocyte reaction and associated energy metabolic changes were assessed by immunostaining, gene expression, proteomics, metabolomics, and extracellular flux analyses after 24 h of exposure of human ReN-derived astrocytes to digoxin (1–10 µM) or TNFα (30 ng/ml) used as a positive control. Strong astrocytic reaction was observed, accompanied by increased glycolysis at low concentrations of digoxin (0.1 and 0.5 µM) and after TNFα exposure, suggesting that increased glycolysis may be a common feature of reactive astrocytes, independent of the triggering molecule. In conclusion, whether astrocyte activation is triggered by cytokines or a xenobiotic, it is strongly tied to energy metabolism in human ReN-derived astrocytes. Increased glycolysis might be considered as an endpoint to detect astrocyte activation by potentially neurotoxic compounds in vitro. Finally, ReN-derived astrocytes may help to decipher mechanisms of neurotoxicity in ascertaining the ability of chemicals to directly target astrocytes.

**Supplementary Information:**

The online version contains supplementary material available at 10.1007/s12035-022-03057-1.

## Introduction

Astrogliosis is a complex cellular process involving astrocytes that is part of the neuroinflammatory cascade of reactions occurring in response to various types of CNS injury [1]. The term astrogliosis dates back to late nineteenth and early twentieth century, when neuroanatomists recognized that astrocytes underwent pronounced structural changes in response to CNS damage and disease [for review, 2]. More recently, a detailed working definition of astrogliosis has been proposed [2] suggesting (i) that astrogliosis is a finely tuned spectrum of changes in astrocytes occurring in response to CNS injury and disease and (ii) that these changes depend on the severity of the insult, are regulated in a context-specific manner by many different signaling molecules, and may alter astrocyte activities both through gain and loss of functions [3]. However, several aspects of astrocyte reaction are still unclear or even controversial, including the denomination of the process [1].

Tight metabolic interactions between astrocytes and neurons have been demonstrated, in particular in terms of energy metabolism [4], defense against oxidative stress [5], and neurotransmitter reuptake and recycling [6]. Also, various modifications in astrocyte metabolism and inflammation levels have been recently reported as a function of age leading to the hypothesis of an age-related interconnection between mitochondrial metabolism and inflammatory responses [7].

Astrogliosis has been reported after exposure to a wide variety of environmental compounds, in various in vitro models [8–11], as well as in animals [12–15]. However, there is still a lack of characterization of this process, in particular in the field of neurotoxicology, where the question remains as to whether astrocytes are directly (primarily) activated by neurotoxins or indirectly (secondarily) through the release of bioactive molecules, such as pro-inflammatory cytokines, by other primarily targeted CNS cell type(s). Furthermore, the modifications of energy metabolism that could potentially occur in astrocytes during their activation by neurotoxins have not yet been investigated. In addition, although astrocyte energy metabolism has been mainly studied in rodent models, very little has been done in human astrocytes.

We have previously shown that upon exposure to pro-inflammatory cytokines (TNFα or IL1β), human astrocytes become activated and in parallel more glycolytic [16]. Here, we hypothesized that changes in energy metabolism of astrocytes will also be coupled to their activation by xenobiotics and not only to activation triggered by cytokines. If this is verified, glycolysis-related proteins and metabolites would allow to widen the panel of molecules used as endpoints to detect neurotoxic compounds.

Digoxin is a well-established and widely used cardiotonic drug [17, 18] that crosses the blood–brain barrier and has been measured in human brains (9–54 ng/g of tissue) [19]. There are clinical evidences that this xenobiotic can induce neuropsychiatric symptoms at therapeutic dose [20], such as headache, hallucinations, convulsions, encephalographic abnormalities, delirium, and drowsiness [21, 22], showing neurotoxic side effects of this drug. In support of this, the neurotoxicity of cardiotonic drugs has been previously shown after intraventricular injections of cardiotonic drugs in cats [23, 24]. In addition, digoxin has been shown to induce astrocyte activation in the complex in vitro 3D aggregating rat brain cell culture system [25].

The aims of the present work were to investigate if xenobiotics could directly induce astrocyte reaction and if this reaction would be accompanied by increased glycolysis. To this end, we exposed human ReN-derived astrocytes to digoxin or to TNFα, chosen as a positive control based on our previous work [16]. We then assessed the reaction and energy metabolism of the exposed astrocytes.

## Material and Methods

### Cell Cultures

ReN cell VM human progenitor (ReN) cells were purchased from Merck. The cells were plated in laminin-coated 75 cm^2^ flasks and maintained in DMEM/F12 medium (Gibco) supplemented with GlutaMax 2 mM (Life Technologies), B27 without vitamin A (Gibco), heparin 1 U/mL (Sigma), bFGF 20 ng/mL (PeproTech), and EGF 20 ng/mL (PeproTech) and kept in an incubator with 5% CO_2_. Medium was renewed three times per week until obtaining 90% confluency. In order to obtain astrocytes, ReN cells were plated onto laminin-coated 6-well plates (5 × 10^5^ cells/well) in ReN medium. The day after, ReN medium was replaced with GMEM (Gibco) with FCS 10% (Gibco). Medium was renewed three times per week for 3 weeks.

### Treatments

**Fig. 1:**
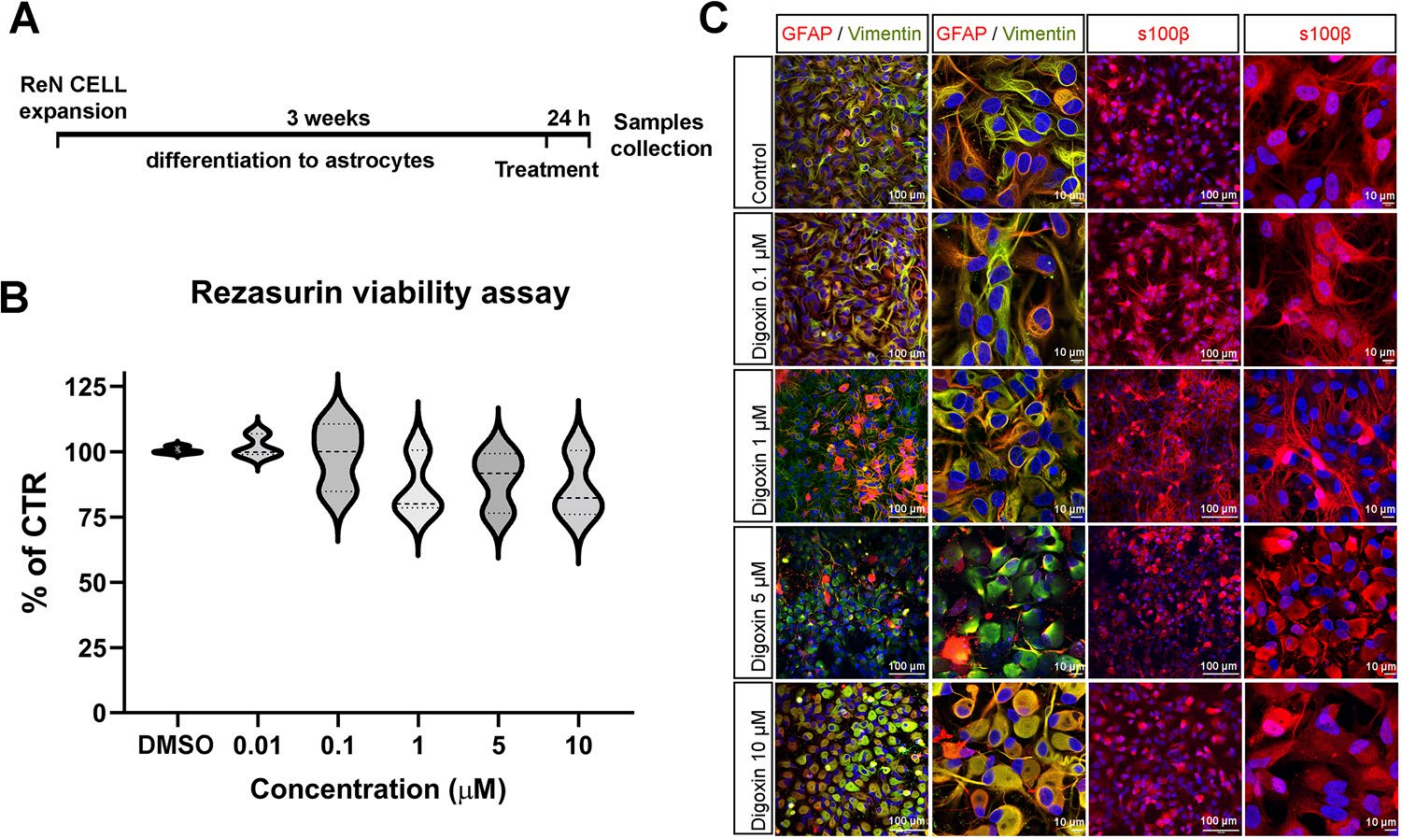
Characterization of Ren-derived astrocytes after digoxin exposure. **A** Diagram of the culture and treatment procedure. **B** Resazurin viability assay for astrocytes exposed to different concentrations of digoxin for 24 h. Results are displayed as violin with data points, for each group *n* = 7 samples obtained in 2 independent experiments. Statistical analysis for the effects of digoxin was performed using the non-parametric Kruskal–Wallis test followed by Dunn’s multiple comparison test. **C** Confocal images of immunostaining for GFAP, vimentin, and S100B a nuclei are stained with Hoechst (blue)

After 3 weeks of differentiation, astrocytes derived from ReN cells were exposed for 24 h to TNFα (30 ng/mL) or different concentrations of digoxin (0–25 µM) (Fig. 1A). Stock solutions were prepared in ultrapure H_2_O for TNFα and DMSO for digoxin. Final DMSO concentration was kept below 0.1%. For exposure of the cells, aliquots of the stock solutions were added to fresh medium to reach the final nominal concentrations indicated above.

### Cell Viability

After treatment, each well was washed once with 1 mL DPBS and then incubated for 1 h at 37 °C with 500 μL of the fluorescent probe resazurin (44 µM final concentration). Resorufin was detected at 540 nm excitation and 590 nm emission wavelengths using a Synergy fluorescence plate reader (BioTeK).

### Immunocytochemistry

Three glass coverslips were placed in each well of a 6-well plate. Then wells were coated with laminin, and 5 × 10^5^ cells were plated per well. Cells were cultured and treated as indicated in “Cell cultures” and “Treatments” sections, respectively. After exposure, cells were fixed for 1 h with 4% paraformaldehyde, and then, coverslips were transferred to a dry surface and stained by adding a drop of the different solutions. First, astrocytes were incubated for 1 h in blocking solution consisting of 5% normal goat serum (NGS) in PBS with 0.4% Triton-X100 (Sigma-Aldrich). Then coverslips were washed 3 times with PBS. Afterwards, samples were incubated for 48 h at 4 °C with a combination of primary antibodies (Suppl. Table 1) diluted 1:200 in PBS containing 3% NGS and 0.1% Triton-X100. After 48 h, samples were washed 3 times for 5 min in PBS by adding and removing a drop of PBS on top of the fixed cells. Samples were further incubated 1 h with secondary antibodies (Suppl. Table 1) diluted in PBS with 3% NGS at room temperature. Subsequently, cells were washed again 3 times for 5 min each with PBS, the nuclei were stained with Hoechst 33342 (1:10,000, Thermo Fisher) for 5 min. Finally, samples were mounted on glass slides by using Immu-mount (Thermo Fisher Shandon Immu-mount). The images were taken using a Zeiss LSM 780 GaAsP confocal microscope.

### Gene Expression

Total RNA was extracted using RNeasy kit (Qiagen,) according to manufacturer’s guidance. RNA concentration was determined by spectrophotometry using NanoDrop ND-1000. Reverse transcription was performed on 1 µg total RNA with the High-Capacity cDNA Reverse Transcription Kit (Life Technologies, US) on a 2720 Thermo Cycler (Applied Biosystems). Real-time PCR analyses were performed using Power SYBR Green (LifeTechnologies, USA) with primers listed in Suppl. Table 2 or Taqman Master Mix with probes referenced in Suppl. Table 3, using a 7900 HT thermocycler (Applied Biosystems). Each sample is analyzed in triplicates. The ΔΔCt method [26] was used to calculate the relative mRNA expression. Data was accepted at < 40 cycles of amplification. Results are expressed as fold change to untreated control cultures maintained under normal medium conditions, set at 1 baseline. Beta-actin (Actb) was used as reference gene.

### Lactate Release

Lactate was measured in the medium after centrifugation (10 min, 300* g*, 4 °C) by a spectrophotometric method. Briefly, 100 µL of medium was added to 100 µL of buffer (glycine-semicarbazide hydrochloride 330 mM, NAD 15 mM, and LDH 70 U/mL) in 96-well plates. Plates were incubated 1 h at 37 °C and then cooled down to room temperature. Absorbance was read at 340 nm on a Synergy plate reader (BioTeK). Results were calculated from a standard curve made with lactate (Sigma-Aldrich).

### Extracellular Flux Analysis

The oxygen consumption rate (OCR, pmoles O_2_ consumed/min) and the extracellular acidification rate (ECAR, mpH/min) were determined using the Seahorse XF96 Extracellular Flux Analyzer (Agilent Technologies). Two kits were used. The Agilent Seahorse XF Cell Mito Stress Test Kit (Agilent Technologies) quantitates the OCR of cells to measure parameters related to mitochondrial function. The Seahorse XF Glycolysis Rate Assay Kit (Agilent Technologies) measures preferentially the glycolytic function in cells. ReN cells were counted and seeded (2 × 10^4^ cells/well) in XF96 Seahorse® microplates precoated with poly-D-lysin (Thermo Fisher Scientific). After 21 days of differentiation (as described above), cells were washed 2 times with XF Assay Medium supplemented with 1 mM pyruvate, 2 mM glutamine, and 10 mM glucose (all from Agilent Technologies). Then 100 µL of the same medium was added in each well. The plate was left to equilibrate for 1 h in a CO_2_-free incubator before being transferred to the Seahorse XF96 analyzer. The pre-hydrated cartridge was filled with the test compounds (for XF Cell Mito Stress Test Kit: oligomycin 2 µM, carbonylcyanide-4-(trifluoromethoxy)phenylhydrazone (FCCP) 2 µM, and rotenone/antimycin A 1 µM each; for XF Glycolysis Rate Assay Kit: rotenone/antimycin A 1 µM each, 2-deoxy-D-glucose 50 mM) and calibrated for 30 min in the Seahorse Analyzer. All the experiments were performed at 37 °C. The Seahorse XF Report Generator automatically calculated the parameters from Wave data that were exported to Excel for further analysis.

### Sample Preparation for Proteomic Analysis

Cell pellets were resuspended in 0.1% RapiGest (Waters) and 100 mM TEAB (Sigma-Aldrich) and sonicated (five cycles of 20 s with breaks, on ice) and incubated for 10 min at 80 °C. Samples were then spun down (14000* g*, 10 min, 4 °C), and the supernatant was recovered. The protein content was measured using Bradford assay (BioRad). For each sample, 10 μg of proteins was reduced using TCEP (final concentration 5 mM, 30 min, 37 °C) (Sigma-Aldrich), alkylated using iodoacetamide (final concentration 15 mM, 60 min, at room temperature, in dark condition) (Sigma-Aldrich), and submitted to an overnight tryptic digestion (w/w ratio 1:50) (Promega). The RapiGest surfactant was cleaved by incubating samples with 1% trifluoroacetic acid (Sigma-Aldrich) (45 min, 37 °C). Samples were then desalted on a C18 reverse phase column (Harvard Apparatus); peptides were dried under vacuum and subsequently resuspended in an appropriate volume of 5% acetonitrile 0.1% formic acid and spiked with iRT peptide (Biognosys, 1:20) for mass spectrometry (MS) analysis.

### MS Data Acquisition for Proteomics Analyses and Data Analysis

For LC–MS/MS, peptides were dissolved in 5% MeCN/0.1% formic acid to a concentration of 0.5 µg/µL. Mass spectrometry experiments were performed by liquid chromatography–electrospray ionization–MS/MS (LC–ESI–MS/MS) in a system consisting of an Easy nLC1200 liquid chromatograph (Thermo Fisher Scientific) and an Orbitrap Fusion Lumos Tribrid mass spectrometer (Thermo Fisher Scientific). Peptides were trapped on an Acclaim pepmap100, C18, 3 μm, and 75 μm × 20 mm nanotrap-column (Thermo Fisher Scientific) and separated on a 75 μm × 500 mm, C18 ReproSil-Pur (Dr. Maisch GmBH), 1.9 μm, 100 Å, home-packed column. Peptides were separated using a 160-min segmented gradient of 0.1% formic acid (solvent A) and 80% acetonitrile 0.1% formic acid (solvent B) (Suppl. Table 4), at a flow rate of 250 nL/min. Data-independent acquisition (DIA) was performed with MS1 full scan at a resolution of 60,000 (FWHM). MS1 was acquired in the Orbitrap with an AGC target of 1 × 10^6^ ions, a maximum injection time of 50 ms, and a scan range from 400 to 1250 m/z followed by 30 DIA MS2 scan with variable windows. DIA MS2 was performed in the Orbitrap using higher-energy collisional dissociation (HCD) at 30%, AGC target of 2 × 10^6^, and a maximum injection time of 54 ms.

The raw DIA MS data were matched against the spectral library following the published protocol [27]. For the data analysis, protein abundances and peptides intensities were exported and analyzed using mapDIA [28]. No further normalization was applied. The following parameters were used: min peptides = 2, max peptides = 10, min correl =  − 1, min_DE = 0.01, max_DE = 0.99, and experimental_design = independent design. Proteins were considered to have significantly changed in abundance with a local false discovery rate LFDR < 0.05 and an absolute fold change (|FC|) > 1.2. Data are available via ProteomeXchange with identifier PXD029370.

### Metabolomics

Metabolomics studies were conducted based on a previously developed multi-platform methodology [29]. Chromatography was performed on a Waters H-Class Acquity UPLC system composed of a quaternary pump, a column manager, and an FTN autosampler (Waters Corporation). For RPLC analyses, samples were separated on a Kinetex C18 column (150 × 2.1 mm, 1.7 μm) and the corresponding SecurityGuard Ultra precolumn and holder (Phenomenex). Solvent A was H_2_O, and solvent B was MeCN, both containing 0.1% formic acid. The column temperature and flow rate were set at 30 °C and 300 µL min^−1^, respectively. The gradient elution was as follows: 2% to 100% B in 14 min, hold for 3 min, then back to 2% B in 0.1 min, and re-equilibration of the column for 7.9 min. Amide-HILIC separations (aHILIC) were conducted on a Waters Acquity BEH Amide column (150 × 2.1 mm, 1.7 µm) bearing an adequate VanGuard precolumn. Solvent A was H_2_O:MeCN (5:95, v/v), and solvent B was H_2_O:MeCN (70:30, v/v) containing 10 mM ammonium formate (pH = 6.5 in the aqueous component). The following gradient was applied: 0% B for 2 min, increased to 70% B over 18 min, held for 3 min, and then returned to 0% B in 1 min and to re-equilibrate the column for 7 min (total run time was 31 min). The flow rate was 500 µL min^−1^, and the column temperature was kept at 40 °C. For the zwitterionic-HILIC (zHILIC) zHILIC method, separation was performed on a Merck SeQuant Zic-pHILIC column (150 × 2.1 mm, 5 μm) and the appropriate guard kit. The following gradient of mobile phase A (MeCN) and mobile phase B (2.8 mM ammonium formate adjusted to pH 9.00) was applied: 5% B for 1 min, increased to 51% B over 9 min, held for 3 min at 51% B, and then returned to 5% B for 0.1 min before re-equilibrating the column for 6.9 min (total run time was 20 min) at a flow rate of 300 µL min^−1^ and a column temperature of 40 °C.

In all cases, a sample volume of 10 µL was injected. Samples were randomized for injection, and QC samples from pooled aliquots of the biological samples were analyzed every six runs to monitor the performance of the analytical platform [30].

The UPLC system was coupled to a maXis 3G Q-TOF high-resolution mass spectrometer from Bruker (Bruker Daltonik GmbH) through an electrospray interface (ESI). The instrument was operated in TOF mode (no fragmentation). The capillary voltage was set at –4.7 kV for ESI + ; drying gas temperature was 225 °C; drying gas flow rate was set at 5.50 (RPLC), 8.00 (aHILIC), or 7.00 (zHILIC) L min^−1^; and nebulizing gas pressure was 1.8 (RPLC) or 2.0 bar (HILIC). Transfer time was set at 40 (RPLC) or 60 (HILIC) µs and pre-pulse storage duration at 7.0 (RP) or 5.0 μs (HILIC). For ESI– operation, the capillary voltage was set at 2.8 kV. All the remaining ion source and ion optics parameters remained as in ESI + . Data between 50 and 1000 m/z were acquired in profile mode at a rate of 2 Hz. ESI and MS parameters were optimized using a mix of representative standards fed by a syringe pump and mixed with the LC eluent (mid-gradient conditions) within a tee-junction. Formate adducts in the 90–1247 m/z range were employed for in-run automatic calibration using the quadratic plus high-precision calibration algorithm provided by the instrument’s manufacturer. MS and UPLC control and data acquisition were performed through the HyStar v3.2 SR2 software (Bruker Daltonik) running the Waters Acquity UPLC v.1.5 plug-in.

Run alignment, peak picking, and sample normalization were performed on Progenesis QI v3.0 (Nonlinear Dynamics, Waters, Newcastle upon Tyne, UK), and peaks were identified by matching their retention times, accurate masses, and isotopic patterns to those of a library of chemical standards (MSMLS, Sigma-Aldrich) analyzed under the same experimental conditions, as described elsewhere [29].

The matrix of features was submitted to noise filtering, analytical drift correction (locally weighted scatter plot smoother (LOWESS)) and sample amount normalization (Probabilistic Quotient Normalization, PQN) by using SUPreMe, an in-house developed software for metabolomics data pre-treatment. Multivariate analysis of the resulting data was conducted on SIMCA 16 64-bit (Umetrics, Sartorius Stedim Data Analytics AB).

### Pathway Enrichment

Pathway enrichment was performed using Metacore software v21.2 (Clarivate Analytics) to match differentially regulated proteins onto biological pathways [31]. The top ten of significantly enriched pathways were studied for several combinations of treatment. List of identified metabolites was also used to analyze significantly enriched pathways.

### Statistical Analysis

Prism 9.3.0 software (GraphPad Software, San Diego, USA) was used for statistical data analysis and graphic representation for gene expression, metabolites, and extracellular flux analyses. Statistical analysis for the effects of digoxin was performed using the non-parametric Kruskal–Wallis test followed by Dunn’s multiple comparison test and for the effects of TNFα using Mann–Whitney test. Statistical test, actual *p* values when significant, and the number of samples are reported in the figure legends. Statistical significance in the figures is indicated as *p < 0.05, ** *p* < 0.01, *** *p* < 0.001, and **** *p* < 0.0001.

## Results

### Selection of the Neurotoxin Used in This Study

Five neurotoxins were tested for their ability to directly induce human astrocyte reactivity in the ReN-derived astrocytes, a single cell-type culture model: the herbicide paraquat, the metals trimethyltin chloride and lead chloride, the mycotoxin ochratoxin A, and the cardiotonic drug digoxin that were all previously shown to induce astrocyte activation in the complex in vitro 3D aggregating rat brain cell culture system [8, 25, 32–34], and some of them also in vivo [14, 35].

ReN-derived astrocytes were exposed during 24 h to paraquat (PQ, 1.25–10 µM), trimethyltin (TMT, 1.25–10 µM), lead chloride (PbCl_2_, 0.1–50 µM), ochratoxin A (OTA, 0.1–50 µM), and digoxin (1–10 µM). Reactivity of astrocytes was evaluated by quantifying the gene expression of glial fibrillary acidic protein (*GFAP*), vimentin (*VIM*), tumor necrosis factor alpha (*TNFα*), interleukin 1 beta (*IL1β*), and interleukin 6 (*IL6*), by qRT-PCR, as well as by immunostaining for GFAP, VIM, and S-100 calcium-binding protein, beta chain (S100B). PQ, TMT, PbCl_2_, and OTA did not modify any of the tested parameters, suggesting an absence of direct effect on ReN-derived astrocytes (results not shown). Digoxin instead affected all parameters tested. This compound was therefore chosen for the present study, and results are shown below.

### Evaluation of the Cytotoxicity of Digoxin on Human Astrocytes

To define non-cytotoxic concentrations for subsequent experiments (in order to work under the level of general toxicity), resazurin viability assay was performed on ReN-derived astrocytes exposed to a range of digoxin concentrations (0, 0.01, 0.1, 1, 5, and 10 µM) during 24 h. A very slight but not statistically significant decrease in astrocyte viability was observed (Fig. 1B). However, no obvious decrease in the number of nuclei (Fig. 1C, blue staining) was observed on the immunocytochemistry, suggesting the absence of overt cytotoxicity at the tested concentrations of digoxin.

### Digoxin and TNFα Share Modifications of the Astrocyte Metabolome and Proteome

To compare the global effects of digoxin and pro-inflammatory cytokines, ReN-derived astrocytes were exposed for 24 h to digoxin (1 and 10 µM) or TNFα (30 ng/ ml). Proteomic and metabolomic analyses were performed for each condition. TNFα (30 ng/ ml) was chosen as a positive control, i.e., a compound shown to activate astrocytes and increase their glycolytic energy metabolism after a 24-h exposure, based on our previous publication [16].

**Fig. 2:**
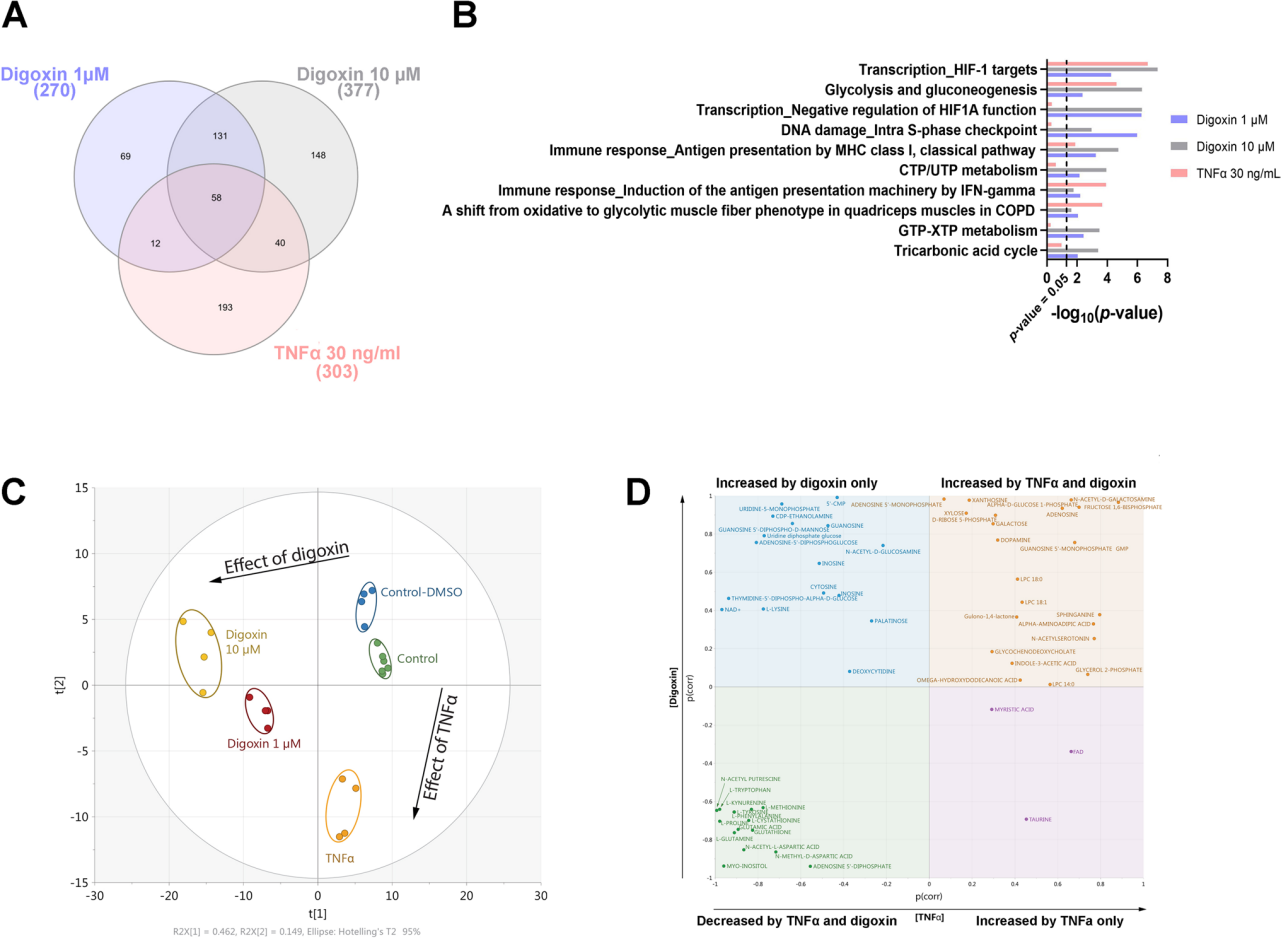
Proteomics and metabolomics profiling of human astrocytes after digoxin and TNFα exposure. **A** Venn diagram of differentially expressed proteins after 24-h exposure to digoxin (1 and 10 µM) or TNFα 30 ng/mL. **B** Top ten enriched biological protein pathways provided by MetaCore™ software from the lists of changing proteins (|FC|> 1.2, *p* value ≤ 0.05, *N* = 3) after exposure to digoxin (1 and 10 µM) or TNFα 30 (ng/mL). The *X*-axis corresponds to − log_10_(*p* value); the *Y*-axis corresponds to the biological pathways. Vertical line represents the *p* value cut-off of 0.05. **C** PCA of metabolites measured by 3 LC–MS approaches, changing after exposure to digoxin (1 and 10 µM) vs control-DMSO or TNFα 30 ng/mL vs control. **D** SUS plot for comparison of metabolomic differences and similarities between the effects of digoxin and TNFα

Almost 3000 proteins were identified for digoxin and TNFα-exposed astrocytes by using data-independent acquisition-based proteomics (Suppl. Table 5). Proteomic analysis retrieved 270 differentially expressed proteins after exposure to 1 µM of digoxin, 377 after digoxin 10 µM, and 303 after TNFα 30 ng/ml (Fig. 2A), of which 58 differentially proteins were common to the three treatments, suggesting some similarities. In order to identify biological pathways impacted by each treatment, pathway enrichment analysis was undertaken. The results showed (Fig. 2B) that among the top ten enriched pathways, five of them exhibited highly significant enrichment *p* value for the 3 treatments (digoxin 1 µM, digoxin 10 µM, and TNFα 30 ng/ml): HIF-1 targets (*p* values = 4.61 × 10^−8^, 5.61 × 10^−5^ and 2.06 × 10^−7^), glycolysis and gluconeogenesis (*p* values = 5.06 × 10^−7^, 4.43 × 10^−3^ and 2.43 × 10^−5^), antigen presentation by MHC class I (*p* values = 1.84 × 10^−5^, 5.70 × 10^−4^ and 1.33 × 10^−2^), induction of the antigen presentation machinery by IFN-gamma (*p* values = 1.73 × 10^−2^, 6.25 × 10^−3^ and 1.21 × 10^−4^), and a shift from oxidative to glycolytic muscle fiber phenotype (*p* values = 2.39 × 10^−2^, 8.81 × 10^−3^ and 2.19 × 10^−4^) (Fig. 2B), whereas negative regulation of HIF1A function was strongly enriched after digoxin exposure (*p* values = 5.08 × 10^−7^ and 5.35 × 10^−7^) but not after TNFα (*p* value = 4.72 × 10^−1^).

**Fig. 3:**
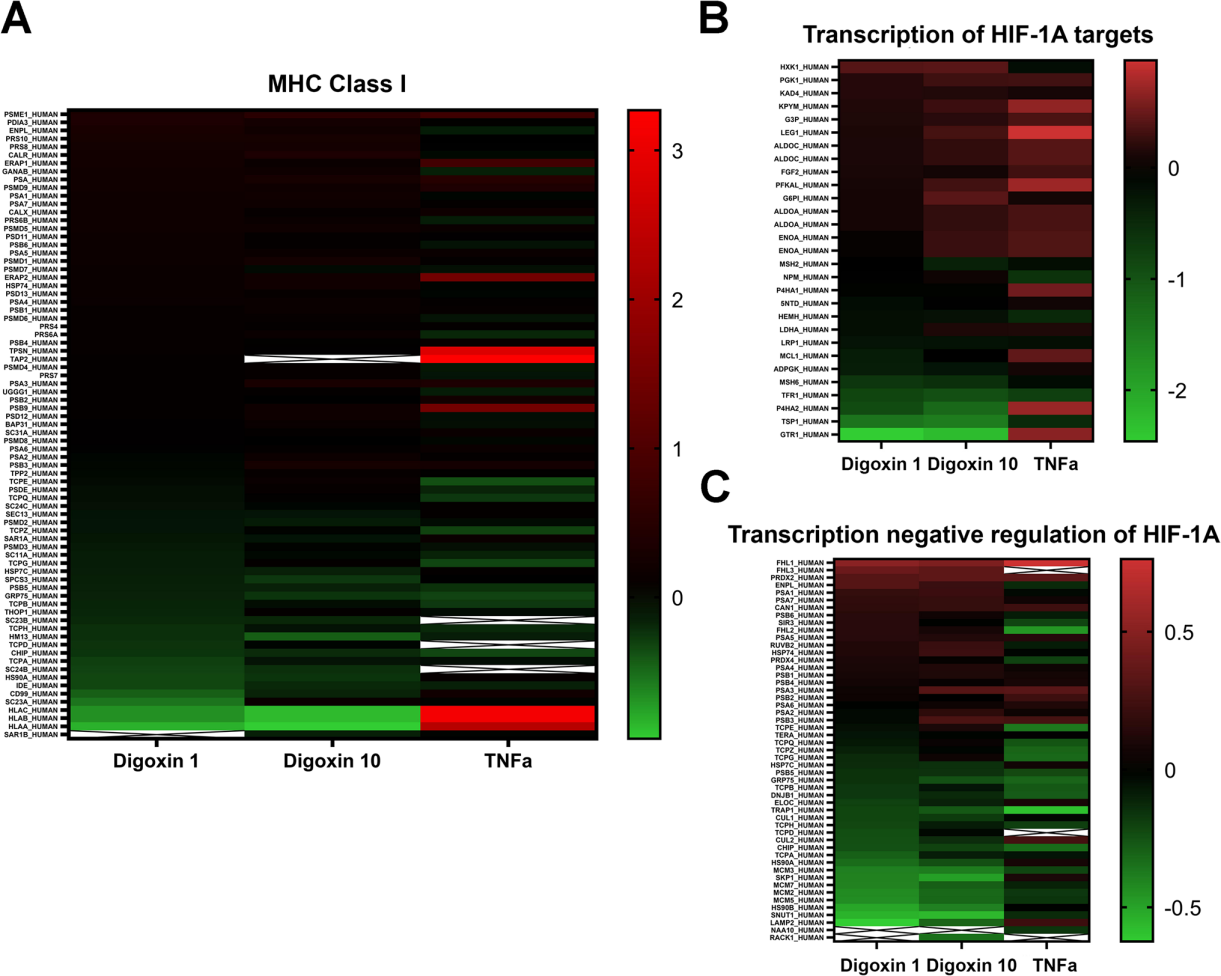
Protein enrichment of inflammatory pathways. Abundance of proteins significantly changed (Log2 FC) after 24 h of exposure to digoxin (1 or 10 µM) or TNFα (30 ng/ml), as compared to control cultures. **A** MHC class I pathway. **B** Transcription HIF-1A targets pathway. **C** Transcription negative regulation of HIF1A pathway

**Fig. 4:**
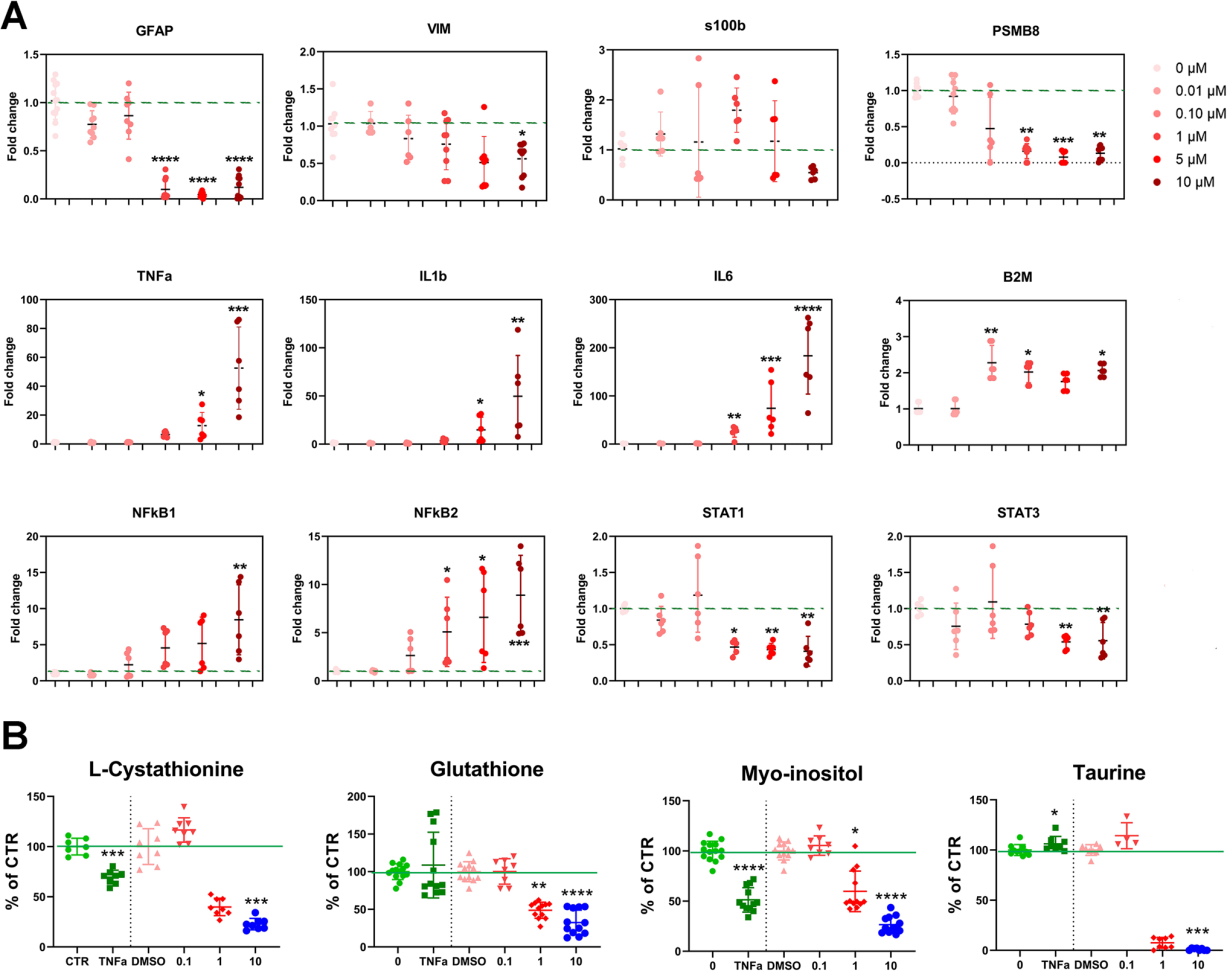
Digoxin-induced human ReN-derived astrocyte reactivity. **A** Relative mRNA levels of genes involved in inflammation after 24 h of exposure to digoxin. For each group *n* = 9–12 samples obtained in 3 independent experiments. **B** Relative levels of metabolites involved in inflammation and cellular volume after 24 h of exposure to digoxin or TNFα. Statistical analysis for the effects of digoxin was performed using the non-parametric Kruskal–Wallis test followed by Dunn’s multiple comparison test and for the effects of TNFα using Mann–Whitney test. *p < 0.05, ** *p* < 0.01, *** *p* < 0.001, **** *p* < 0.0001

Multivariate analysis of metabolomics data exhibited well-differentiated trajectories for digoxin and the reference compound TNFα (Fig. 2C). The metabolic profile induced by digoxin is separated from the control-DMSO on the first principal component (PC) of the PCA analysis with, as expected, the highest concentration of digoxin inducing a stronger alteration. Interestingly, the effect of TNFα appears orthogonal to that of digoxin as shown by its separation from the control group on the second PC of the multivariate model. Two supervised models were then built to specify which metabolic changes are common or exclusive to each substance. A first OPLS-DA model was computed to compare TNFα and its control group, while another OPLS regression model included the two concentrations of digoxin and their DMSO control group. The predictive components of both models were subsequently combined into a shared-and-unique-structures (SUS) plot for joint analysis (Fig. 2D). Such a representation allows for a straightforward visualization of the effects of both treatments highlighting common and/or specific metabolic patterns. The bottom-left and the top-right quadrants show the common metabolites which were decreased and increased, respectively, upon treatment with each of the two molecules. On the contrary, the top-left and bottom-right quadrants contain metabolic features showing alteration patterns specifically due to TNFα or to digoxin. Details on these metabolites are given in the next chapters (Figs. 3B and 4E).

### Digoxin Induced an Inflammatory Response in Human ReN-Derived Astrocytes

To compare the reactivity induced by cytokine or neurotoxin, ReN-derived astrocytes were exposed for 24 h to digoxin (1 and 10 µM) or TNFα (30 ng/ ml). Further detailed analysis of their proteome and metabolome (globally presented in the above section) was performed for each condition. Immunostaining and gene expression analyses were performed for digoxin samples.

Exposure to digoxin induced a significant decrease in the astrocytic cytoskeleton markers glial fibrillary acidic protein (*GFAP*, 1–10 µM) and vimentin (*VIM*, 10 µM) gene expression (Fig. 3A), whereas it did not affect S100B mRNA level. A modification of the cytoskeleton was confirmed by immunocytochemistry for GFAP, VIM, and S100B, showing a shortening of the astrocytic processes and an enlargement of cell bodies (Fig. 1C) (5 and 10 µM). However, no modification in the amount of these proteins was observed by proteomic analysis.

Digoxin also induced a strong and significant upregulation of the mRNA levels of the inflammation markers tumor necrosis factor alpha (*TNFα*), interleukin 1β (*IL1β)*, interleukin 6 (*IL6*), beta-2 microglobulin (*B2M*), and of the proteasome subunit beta type-8 (*PSMB8*) (Fig. 4A). Furthermore, it induced the upregulation of nuclear factor kB subunit 1 and 2 (*NFkB1, NFkB2*), associated with astrocyte detrimental pathway [36] (Fig. 4A). On the other side, signal transducer and activator of transcription 3 (*STAT3*), associated with astrocyte protective pathway, and *STAT1* were statistically significantly downregulated (Fig. 4A).

Strong modifications in the major histocompatibility complex (MHC) class I pathway were revealed by proteomics (Fig. 2B and Fig. 3A). However, although TNα and induced the upregulation of human leukocyte antigen A (HLA-A), HLA-B, and HLA-C, digoxin decreased their expression. The same was observed for antigen peptide transporter 2 (TAP2) and TPSN, both involved in antigen processing. A number of metabolites related with inflammatory and redox processes were also found deregulated. For example, L-cystathionine and myo-inositol showed a significant decrease upon exposure to digoxin (1 and 10 µM) and TNFα (Fig. 4B), whereas glutathione, a well-known anti-oxidant and very important component in many astrocytic detoxification processes [37], and taurine were decreased only by digoxin.

In conclusion, gene expression, immunostaining, proteomics, and metabolomics data together suggest that digoxin induces ReN-derived astrocytes reactivity and inflammatory response.

### Energy Metabolism Is Modified in Astrocytes Activated by Digoxin

To compare the energy metabolism in astrocytes induced by cytokine or neurotoxin, ReN-derived astrocytes were exposed for 24 h to digoxin (1 and 10 µM) or TNFα (30 ng/ ml). Further detailed analysis of their proteome and metabolome (globally presented above) was performed for each condition. Furthermore, gene expression, oxygen consumption, medium acidification, and lactate release were measured after exposure to digoxin.

**Fig. 5:**
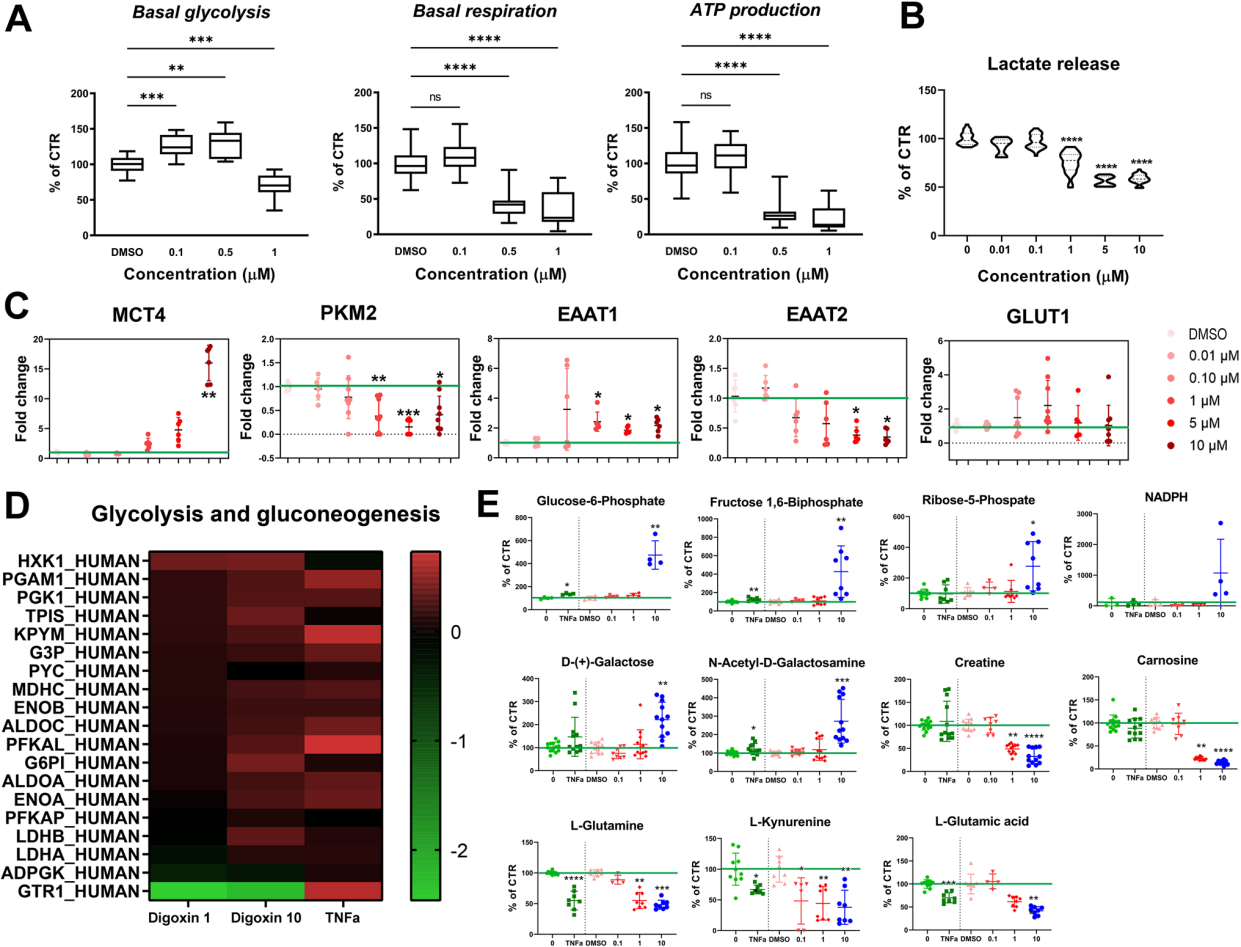
Digoxin-induced changes in energy metabolism. **A** Basal glycolysis rate, basal respiration, and ATP production after digoxin (0.1 (*n* = 31), 0.5 (*n* = 14) and 1 µM (*n* = 36)). **B** Lactate release after 24 h of exposure to digoxin. **C** Relative mRNA levels of genes involved in energy metabolism after 24 h of exposure to digoxin. For each group, *n* = 9–12 samples obtained in 3 independent experiments. **D** Heatmap of significantly changed proteins (Log2 FC) of the glycolysis pathway after 24 h of exposure to digoxin (1 and 10 µM) or TNFα (30 ng/mL). **E** Relative levels of metabolites involved in energy metabolism after 24 h of exposure to digoxin or TNFα. Statistical analysis for the effects of digoxin was performed using the non-parametric Kruskal–Wallis test followed by Dunn’s multiple comparison test and for the effects of TNFα using Mann–Whitney test. *p < 0.05, ** *p* < 0.01, *** *p* < 0.001, **** *p* < 0.0001

Seahorse data showed a significant increase in glycolysis after exposure to digoxin 0.1 and 0.5 µM (Fig. 5A) that was followed by a significant decrease at 1 µM. In parallel, a significant decrease in basal respiration and ATP production was observed at 0.5 and 1 µM of digoxin and a decrease in lactate release from 1 to 10 µM (Fig. 5B).

The gene expression of monocarboxylate transporter 4 (*MCT4*), specifically expressed by astrocytes, was upregulated after digoxin treatment but statistically significantly only at 10 µM (Fig. 5B), whereas the expression of pyruvate kinase M2 isoform (*PKM2*), an enzyme that catalyzes the final irreversible step in glycolysis, was statistically decreased. Digoxin exposure also induced an increase of the excitatory amino acid transporter 1 (*EAAT1*) and a decrease in EAAT2 mRNA levels (Fig. 5C). The gene expression of glucose transporter 1 (*GLUT1*), the main glucose transporter, was not affected, although a decrease was observed at the protein level (Fig. 5D) (GTR1).

Glycolysis and gluconeogenesis pathway was the second most modified pathway in proteomics analysis after exposure to digoxin (Fig. 2B). A heatmap (Fig. 5D) shows that most of the proteins related to these pathways were slightly upregulated after digoxin or TNFα exposure, such as pyruvate kinase protein (KPYM), a key enzyme in glycolytic pathway [38], and phosphofructokinase (PFKAL) that catalyzes the phosphorylation of D-fructose 6-phosphate to fructose 1,6-bisphosphate by ATP, the first committing step of glycolysis or glyceraldehyde-3-phosphate dehydrogenase (G3P) also a key enzyme in glycolysis [39] which, in addition, has a function in innate immunity by enhancing TNF-induced NFκB activation and type I interferon production [40]. The only protein of this pathway that was not regulated in the same direction by digoxin and TNFα is glucose transporter 1 (GTR1) that was strongly downregulated by digoxin whereas upregulated by TNFα. Analysis of the proteome also showed that digoxin and TNFα alter two pathways related to hypoxia-inducible factor (HIF): transcription of HIF-1 targets and negative regulation of HIF-1A (Fig. 2B and Fig. 3B and C). HIF is a pivotal regulator of glucose metabolic process, shifting cellular energy metabolism from oxidative phosphorylation to canonical glycolysis that activates the transcription of GTR1 [41].

Several metabolites related to energy metabolism were found altered after exposure to digoxin and TNFα. Glucose-6-phosphate and fructose 1,6-bisphosphate were increased by both treatments, whereas ribose-5-phosphate was increased by digoxin but not by TNFα (Fig. 4E). The level of NADPH was highly increased at 10 µM of digoxin, however not statistically significantly due to the high variability generated by the highest point. These 4 metabolites are involved in the pentose phosphate pathway. D-( +)-galactose and its derivative N-acetyl-D-galactosamine increased after digoxin exposure, whereas creatine and carnosine decreased [42]; only N-acetyl-D-galactosamine was very slightly increased by TNFα treatment. Finally, the level of L-glutamine, L-kynurenine, and L-glutamic acid decreased after digoxin and TNFα.

Altogether these data suggest that digoxin modifies energy metabolism of astrocytes, possibly as a consequence of their activation, as already reported for pro-inflammatory cytokines [16].

## Discussion

Astrocytes, fulfilling numerous extremely important roles in CNS, have long been neglected in neurotoxicology. However, they have the versatility to endorse a continuum of neuroprotective to neurodegenerative actions that make them very interesting as markers of neurotoxicity. In the field of toxicology, there is nowadays a large consensus to replace animal testing by new alternative methods (NAMs), not only for ethical and economic arguments but also for scientific reasons [43]. Also, the in vitro NAMs should favor the use of human cells. This is of importance for the present study since some observed species differences in the astrocytes-to-neuron ratio, astrocyte morphology, and gene expression profile have led to the hypothesis that the regulation of astrocytic functions may differ between humans and rodents [44]. Here, ReN cell-derived astrocytes were chosen for their very high reproducibility and robustness [16].

The upregulation of GFAP has long been recognized as the hallmark of reactive astrocytes and early proposed as a sensitive marker of neurotoxicity [8, 9, 45–47]. Although this still stands true, other modifications of astrocyte cytoskeleton, including the decrease of GFAP, as well as other cytoskeletal proteins, such as vimentin and S100B, could now also be considered [16, 34, 48, 49]. In the present study, digoxin profoundly affected the cytoskeleton and the inflammatory response of human astrocytes, in the absence of overt cytotoxicity. GFAP, vimentin, and S100B gene expression was downregulated, and immunostaining showed shortening of astrocytic processes, as previously reported after the exposure of the same cells to the pro-inflammatory cytokines TNFα or IL1β [16] or after exposure of a multi-cellular rat 3D cell culture system to ochratoxin A [34]. Among the five xenobiotics preliminary tested for this study, digoxin was the only one to trigger modifications of the astrocytic cytoskeleton and upregulation of pro-inflammatory cytokines mRNAs, showing its ability to directly (primarily) trigger astrocyte reaction. This suggests that the activation of astrocytes by paraquat, trimethyltin chloride, lead chloride, or ochratoxin A, observed in complex in vitro cultures containing various brain cell types [8, 25, 32–34], was indirect (secondary) and occurred via the primary damaging action of the chemicals on another brain cell type (most of the time the neurons) leading to the release of bioactive molecules, such as pro-inflammatory cytokines.

Next to cytoskeleton changes, digoxin induced the expression of transcription factors and genes involved in the inflammatory response, i.e., NFκB1, NFκB2, TNFα, IL1β, IL6, STAT1, and STAT3 [36], as previously described after treatment of the same type of cells with pro-inflammatory cytokines [16], again showing that digoxin was able to directly induce the reaction of human astrocytes in vitro. Nevertheless, the activation of astrocytes induced by digoxin was not exactly the same as the one induced by cytokines, since digoxin decreased STAT1 and STAT3 gene expression, while pro-inflammatory cytokines increased STAT1 and did not affect STAT3. The inflammatory status of ReN-derived astrocytes exposed to digoxin is also highlighted by the presence of two immune response pathways, MHC class I and antigen presentation by IFN-gamma, in the top ten enriched pathways found after analysis of proteomics data. These two pathways were also enriched after TNFα in this study and also after exposure to IL1β [16]. It has been shown recently that B2M, a molecule of the MHC-I pathway, is required for the development of astrogliosis. Therefore, the increase in B2M gene expression observed after digoxin, and previously reported after exposure to TNFα [16], further supports the fact that digoxin induces astrocyte activation. On the other hand, HLA-A, HLA-B, and HLA-C are modified in the opposite way by TNFα and digoxin, TNFα upregulating HLA proteins whereas digoxin downregulating their expression. In line with a recent study indicating that besides playing a role in the immune response, HLA class I antigens are crucial to sustain glycolysis in melanoma cells [50], TNFα was shown to induce glycolysis in ReN-derived astrocytes [16]. In the present study, the regulation of HLA class I proteins was not studied at concentrations where digoxin stimulates glycolysis. Thus, besides both exposure with pro-inflammatory cytokines and digoxin induced astrocyte activation, some markers of inflammation showed different tendency; however, this divergence might not be found at lower concentration of digoxin.

Energy metabolism of human ReN-derived astrocytes was modified by digoxin. At the lowest concentration of digoxin (0.1 µM), a stimulation of basal glycolysis was observed by extracellular flux analysis, without change in basal respiration, whereas at 0.5 µM digoxin, increased glycolysis was accompanied by a parallel decrease in basal respiration and ATP, suggesting that reactive astrocytes are reinforcing their glycolytic metabolism, as already reported after exposure to pro-inflammatory cytokines [16]. In addition, proteomics analysis evidenced a slight digoxin-induced upregulation of hexokinase 1 (HXK1) and pyruvate kinase (KPYM), the key enzymes in glycolysis. However, at higher but still non-cytotoxic concentrations (from 1 µM), digoxin decreased glycolysis and lactate release. Altogether, these data suggest that digoxin induced an increase in glycolysis in astrocytes that was counteracted at higher concentrations by the strong downregulation of the glucose transporter 1 (GTR1), preventing an efficient cellular uptake of glucose. Indeed, the raise in glycolysis previously reported after treatment of human ReN-derived astrocytes with TNFα or IL1β was accompanied by an increased *GLUT1* gene expression [16], confirmed in the present study by the increased level of the protein (GTR1) observed in proteomics analysis after TNFα treatment. The decrease in GTR1 level after digoxin exposure may be attributed to the negative regulation of hypoxia-inducible factor (HIF) 1 A pathway. This would be in line with the digoxin-induced inhibition of HIF1α protein expression and of its target genes GLUT1 and HK1 observed in a reporter cell line [51]. The growing literature on HIF shows its importance as a key regulator of immune cell function [52]. HIF1α plays an important role in astrocytes reaction [53]; however here, its beneficial functions might be prevented by the digoxin-induced downregulation of its pathway.

The level of various metabolites, involved both in inflammatory/oxidative stress processes and in energy metabolism, was modulated by digoxin and TNFα. For example, taurine and myo-inositol are strongly involved in the osmotic/volume regulation of brain cells [54, 55], and their modulation during astrocyte activation may be associated to the morphological changes observed in the astrocytes. In addition, taurine is also involved in energy metabolism [56, 57]. Then, the increased levels of glucose-6-phosphate, fructose 1,6-bisphosphate and ribose-5-phosphate after exposure to TNFα and digoxin suggest a stimulation of the pentose phosphate pathway that is a major source of NADPH for resistance to oxidative stress and therefore to inflammation. No statistically significant increase of NADPH was evidenced. However, the trend is very clear considering the fact that a high variability is expected for such an oxidation-sensitive molecule. All these metabolites may be considered as bridges between inflammation and energy metabolism.

Taken together, the results of this study show that human ReN-derived astrocytes are directly activated by digoxin and increase their glycolytic capacity at low concentration without increasing oxidative phosphorylation. The same astrocytic behavior was previously reported after exposure of the same human astrocytes to pro-inflammatory cytokines (TNFα and IL1β), suggesting increased glycolysis as a common response of reactive astrocytes. Knowing the potentially deleterious effects of reactive astrocytes on neurons, our results may explain, at least partially, some of the described neurotoxic side effects reported by patients under digoxin treatment [20, 21].

In conclusion, whether astrocyte activation is triggered by cytokines or a xenobiotic, it is strongly tied to energy metabolism in human ReN-derived astrocytes. Furthermore, the interest of using increased glycolysis and related molecules as new endpoints to detect astrocyte activation in vitro, and therefore identify potentially neurotoxic chemicals, should be further investigated. Finally, ReN-derived astrocytes may help to decipher mechanisms of neurotoxicity in ascertaining the ability of chemicals to directly target astrocytes.

## Supplementary Information

Below is the link to the electronic supplementary material.

Supplementary file1 (DOCX 14 KB)

Supplementary file2 (DOCX 22 KB)

Supplementary file3 (DOCX 21 KB)

Supplementary file4 (DOCX 14 KB)

Supplementary file5 (XLSX 378 KB)

## Data Availability

The data that support the findings of this study are available from the corresponding author upon request.
