# Supplementary material for: Digoxin Induces Human Astrocyte Reaction In Vitro
Source: Mol Neurobiol. 2022 Oct 12;60(1):84–97. doi: 10.1007/s12035-022-03057-1 (PMC9758102; doi:10.1007/s12035-022-03057-1)
Supplement: Supplementary file 1 — Supplementary file1 (DOCX 14 KB) [file 12035_2022_3057_MOESM1_ESM.docx]

**Suppl. Table 1:** List of primary and secondary antibodies used for immunohistochemistry

| **Name** |  | **Species** | **Type** | **Dilution** | **Ref** |
| --- | --- | --- | --- | --- | --- |
| GFAP | Primary | Anti-Rabbit | Polyclonal | 1 :200 | G9269 Sigma |
| S100β | Primary | Anti-Rabbit | Polyclonal | 1 :200 | Z0311 Dako |
| VIMENTIN | Primary | Anti-Mouse | Monoclonal | 1 :200 | AB8978 Abcam |
| ALEXA 488 | Secondary | Donkey anti-mouse | Polyclonal | 1 :200 | A11037 Invitrogen |
| ALEXA 594 | Secondary | Goat anti-rabbit | Polyclonal | 1 :200 | A21202 Invitrogen |
