# Supplementary material for: Digoxin Induces Human Astrocyte Reaction In Vitro
Source: Mol Neurobiol. 2022 Oct 12;60(1):84–97. doi: 10.1007/s12035-022-03057-1 (PMC9758102; doi:10.1007/s12035-022-03057-1)
Supplement: Supplementary file 2 — Supplementary file2 (DOCX 22 KB) [file 12035_2022_3057_MOESM2_ESM.docx]

**Suppl. Table 2:** Sequences of qPCR primers used with SYBR green

| **Gene** | **Forward primer (5’-3’)** | **Reverse primer (5’-3’)** |
| --- | --- | --- |
| *ACTB* | AAATCTGGCACCACACCTTC | AGAGGCGTACAGGGATAGCA |
| *B2M* | TGCTGTCTCCATGTTTGATGTATCT | TCTCTGCTCCCCACCTCTAAGT |
| *GFAP* | CACCACGATGTTCCTCTTGA | GTGCAGACCTTCTCCAACCT |
| *GLUT1* | GGCATTGATGACTCCAGTGTT | ATGGAGCCCAGCAGCAA |
| *IL1β* | TTCGACACATGGGATAACGAGG | TTCGACACATGGGATAACGAGG |
| *IL6* | CCTGAACCTTCCAAAGATGGC | TTCACCAGGCAAGTCTCCTCA |
| *MCT4* | CCATGCTCTACGGGACAGG | GCTTGCTGAAGTAGCGGTT |
| *PKM2* | GCCTGCTGTGTCGGAGAAG | CAGATGCCTTGCGGATGAATG |
| *PSMB8* | CACGCTCGCCTTCAAGTTC | AGGCACTAATGTAGGACCCAG |
| *TNFa* | GAGGCCAAGCCCTGGTATG | CGGGCCGATTGATCTCAGC |
| *VIM* | ATTCCACTTTGGGTTCAAGG | CTTCAGAGAGACGAAGCCGA |
| *S100b* | ATGTCTGAGCTGGAGAAGGC | TTCAAAGAGCTGGTGGCAGG |
