# Supplementary material for: Digoxin Induces Human Astrocyte Reaction In Vitro
Source: Mol Neurobiol. 2022 Oct 12;60(1):84–97. doi: 10.1007/s12035-022-03057-1 (PMC9758102; doi:10.1007/s12035-022-03057-1)
Supplement: Supplementary file 3 — Supplementary file3 (DOCX 21 KB) [file 12035_2022_3057_MOESM3_ESM.docx]

**Suppl. Table 3:** References of Taqman assays

| **Gene** | **Assay ID (Thermofisher)** |
| --- | --- |
| *ACTB* | Hs01060665_g1 |
| *NFkB1* | Hs00765730_m1 |
| *NFkB2* | Hs01028890_g1 |
| *STAT1* | Hs01013996_m1 |
| *STAT3* | Hs00374280_m1 |
| *EAAT1 (SLC1A3)* | Hs00904823_m1 |
| *EAAT2 (SLC1A2)* | Hs01102423_m1 |
