# Supplementary material for: Digoxin Induces Human Astrocyte Reaction In Vitro
Source: Mol Neurobiol. 2022 Oct 12;60(1):84–97. doi: 10.1007/s12035-022-03057-1 (PMC9758102; doi:10.1007/s12035-022-03057-1)
Supplement: Supplementary file 4 — Supplementary file4 (DOCX 14 KB) [file 12035_2022_3057_MOESM4_ESM.docx]

**Supplementary Table 4:** Liquid chromatography gradient

| **%B** | **Time (min)** | **Duration (min)** |
| --- | --- | --- |
| 8 | 0 | 0 |
| 28 | 110 | 110 |
| 42 | 135 | 25 |
| 95 | 140 | 5 |
| 95 | 160 | 20 |
